# Supplementary material for: Lipid composition effects on the number and size of liposomes formed by the inverted emulsion method
Source: Biophys J. 2025 Dec 11;125(3):731–44. doi: 10.1016/j.bpj.2025.12.013 (PMC13351457; doi:10.1016/j.bpj.2025.12.013)
Supplement: Document S1. Figures S1 and S2 [file mmc1.pdf]

Biophysical Journal, Volume *125*

Supplemental information

Lipid composition effects on the number and size of liposomes formed by the inverted emulsion method

Hibiki Sakata, Hitomi Matsubara, Kanako Gomi, and Makito Miyazaki

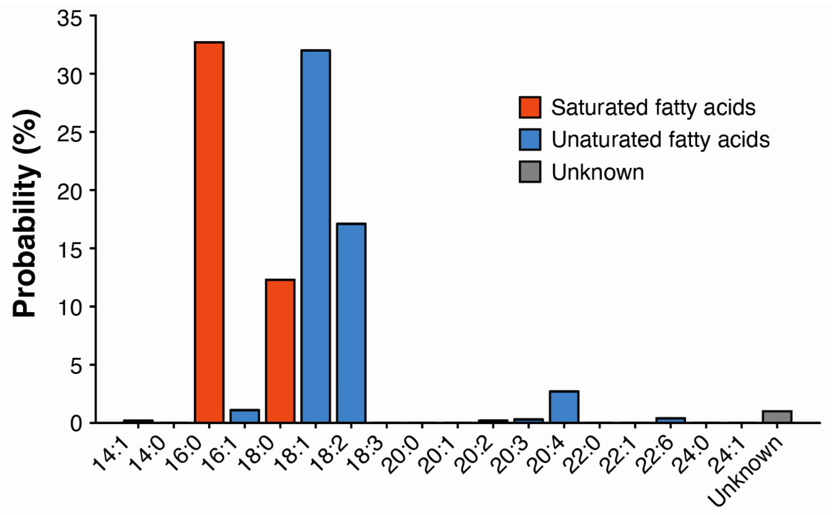

Figure S1. Fatty acid distribution of egg PC.

The analysis data were downloaded from the webpage of Avanti Polar Lipids, Inc. Based on the fatty acid distribution, the mean molecular weight of the lipids was estimated to be 770.123 Da.

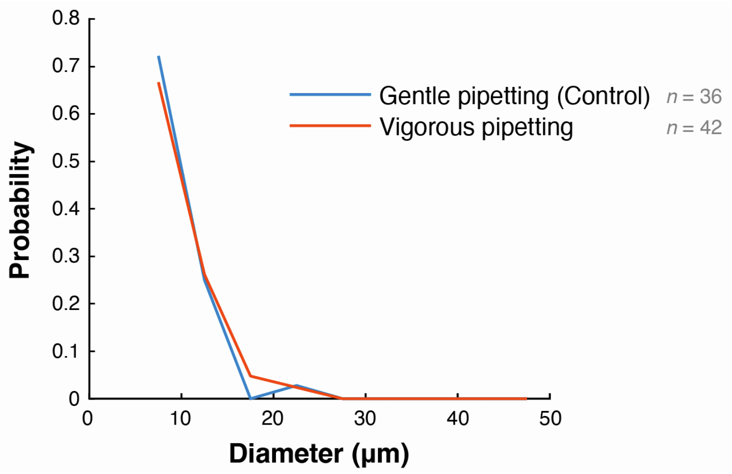

Figure S2. Effects of pipetting on the size distribution of liposomes.

Distribution of liposome diameter. All liposomes were prepared from a lipid-oil mixture containing 90% egg PC and 10% DOPA. The blue line indicates the size distribution of liposomes as a control collected after 30 pipetting strokes at a rate of 1 stroke/sec, by aspirating and dispensing 150 μl of the sample using a 200 μl pipette tip. The red line indicates the size distribution of liposomes collected from the remaining solution of the same sample, after an additional 30 pipetting strokes at a rate of 3 strokes/sec using the same pipette tip and aspiration volume. Liposomes larger than 5 μm in diameter were analyzed. Three independent experiments were performed for each condition.
